# Histological analysis of the repair of dural lesions with silicone mesh in rats subjected to experimental lesions

**DOI:** 10.1590/S1679-45082015AO3378

**Published:** 2015

**Authors:** Fernando William Figueiredo da Rosa, Pedro Henrique Isoldi Pohl, Ana Maria Amaral Antônio Mader, Carla Peluso de Paiva, Aline Amaro dos Santos, Bianca Bianco, Luciano Miller Reis Rodrigues

**Affiliations:** 1 Faculdade de Medicina do ABC, Santo André, SP, Brazil.

**Keywords:** Dura Mater, Meninges, Surgical procedures, operative, Thoracic surgery, Silicon, Models, animal

## Abstract

**Objective:**

To evaluate inflammatory reaction, fibrosis and neovascularization in dural repairs in Wistar rats using four techniques: simple suture, bovine collagen membrane, silicon mesh and silicon mesh with suture.

**Methods:**

Thirty Wistar rats were randomized in five groups: the first was the control group, submitted to dural tear only. The others underwent durotomy and simple suture, bovine collagen membrane, silicon mesh and silicon mesh with suture. Animals were euthanized and the spine was submitted to histological evaluation with a score system (ranging from zero to 3) for inflammation, neovascularization and fibrosis.

**Results:**

Fibrosis was significantly different between simple suture and silicon mesh (p=0.005) and between simple suture and mesh with suture (p=0.015), showing that fibrosis is more intense when a foreign body is used in the repair. Bovine membrane was significantly different from mesh plus suture (p=0.011) regarding vascularization. Inflammation was significantly different between simple suture and bovine collagen membrane.

**Conclusion:**

Silicon mesh, compared to other commercial products available, is a possible alternative for dural repair. More studies are necessary to confirm these findings.

## INTRODUCTION

Spinal trauma and iatrogenic lesions are the most common causes for dural lesions.^(1-5)^ Vertebral fractures can cause dural lesions,^(3-5)^ in up to 19% of cases, when posterior spinal elements have also fractures associated to vertebral body explosion fractures.^(4,5)^ Accidental lesion of the dura mater in lumbar procedures has a prevalence between 1 to 17%.^(1,2)^ Several autologous structures and synthetic materials have been used in dura mater repair. That includes autologous fat, fascia lata, lyophilized dura mater, and synthetic materials with great variability in clinical results.^(6-8)^

The increasing indications and the complexity of spine surgical procedures have led to an increased prevalence of dural lesions,^(3,9)^ particularly in reoperation procedures.^(1,3)^ The lack of attention to the principles of modern surgery, the complexity of the cases, lack of experience, and the rush to complete the surgical procedure are factors that could contribute to this statistic. In the United States, the increase in the number of lumbar laminectomies performed for degenerative diseases of the intervertebral discs has been the leading cause of dural lesions, frequently occurring by the time of opening the ligamentum flavum.^(10)^

Most lesions are recognized intraoperatively. In some cases, in which the lesion is incomplete, affecting only the dura mater external layer without reaching the arachnoid, cerebrospinal fluid may not leak immediately and still occur postoperatively.^(3)^ According to Wang et al., immediate intraoperative repair does not increase perioperative morbidity or affect the outcomes.^(1)^

In dura mater repair surgeries, prevention of postoperative adhesions is an important aspect.^(9)^ Lyophilized dura mater from human cadavers is often used as a dural substitute, and also in cases of repair of dural defects and meningomyeloceles, but the disadvantage of the transmission of infectious diseases exists.^(9)^

Dural repair increases around 20 to 30 minutes to the total surgery time.^(1)^ The proper technique should be quick and easy to apply,^(3)^ however, lesions in the anterior or lateral regions may be more complicated, requiring other types of techniques.^(3,4)^ A combination of methods has been advocated by some authors.^(1-3)^

Dural lesions have been treated successfully through primary repair followed by bed rest in dorsal decubitus.^(1,3,4)^ Various forms of repair have been used, such as sutures with separate stitches,^(1,5)^ continuous sutures,^(3)^ autologous fascia grafts,^(3)^ fibrin sealant,^(11)^ and porcine collagen matrix.^(12)^ Silicon mesh has been used in various surgical fields, we developed a silicon membrane in order to evaluate the applicability of the dural lesion.

It is very difficult to compare the different types of treatment of dural lesion because the few studies that were conducted are based on a small number of patients.^(1)^ This study is therefore important due to the lack of consensus in the international literature about the repair methods and the new materials that are emerging for its treatment. Moreover, experimental controlled studies are rare and would help compare crucial aspects, such as inflammation, biocompatibility, neovascularization and fibrosis between distinct dural repair methods.

## OBJECTIVE

To evaluate the potential of silicone mesh use in the repair of dura mater lesions comparing different techniques.

## METHODS

This experimental study with rats was conducted in the Animal Experimentation Laboratory and Bioterium of the *Faculdade de Medicina do ABC* (FMABC) in accordance with the due legislation. It was approved by the FMABC Animal Experimentation Ethics Committee (protocol number 003/2010).

Thirty male Wistar rats (*Rattus norvergicus*), at least 12 week-old and weighing between 300 and 350g, were used. They were placed in groups of up to three per cage. They were maintained under environmental conditions (handling, *ad libitum, *periodic changing of shavings and cage cleaning), at a controlled temperature of around 28^o^C, with 12 hour light/dark periods and regular daily feeding with food appropriate for the species, until the end of the experiment.

The rats were evaluated for overall condition, weight, and motor skills. The following exclusion criteria were established: death following the lesion; loss of tissue in the area of the lesion; abnormalities observed macroscopically in the medullary region; lack of anal or vesical sphincter control; absence of motor activity following the lesion; and weight loss of more than 20% of the animal’s weight.

The rats were randomly separated (by draw) into five groups composed of six animals each and identified on their tails. All were submitted to lesion of the dura mater. The groups were the following: Durotomy Group, control, with rats submitted to dural lesion without subsequent treatment; Simple Suture Group, with dural lesion repaired with simple sutures; DuraGen^®^ Group, with dural lesion repaired with bovine collagen dural substitute (DuraGen^®^) as a protector, without fixation; Mesh Group, with dural lesion repaired with silicone mesh as a protector, without fixation; Mesh Plus Suture Group, with dura mater lesion repaired with simple sutures and silicone mesh as a protector.

### Description of the surgical technique

Intraperitoneal anesthesia was used (a solution of ketamine, 50 to 80mg/kg, with xylazine chlorhydrate 2%, 10mg/kg, and atropine, 0.05mg/kg). Deep anesthesia was confirmed by the absence of a corneal reflex and the absence of a reaction to deep pain inflicted by interdigital compression of the paws. Subcutaneous cefalotin (25mg/kg) was used for prophylactic antibiotic therapy following the anesthesia, 24 and 48 hours after creating the lesion. In rats that presented infection, the treatment was extended until the tenth day, and in cases of persistent infection, the animal was euthanized (exclusion criterion).

**Figure 1:**
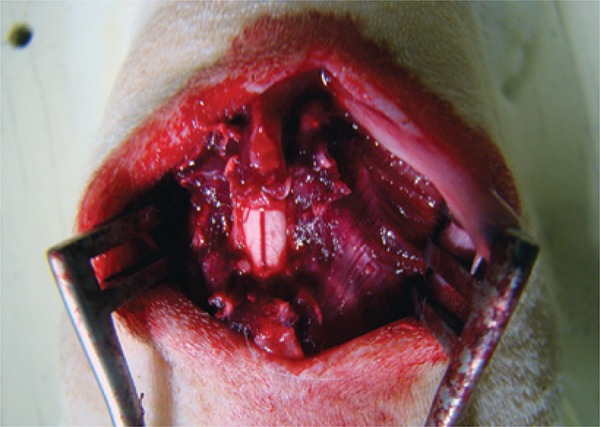
Surgical exposure of the spinal cord

**Figure 2:**
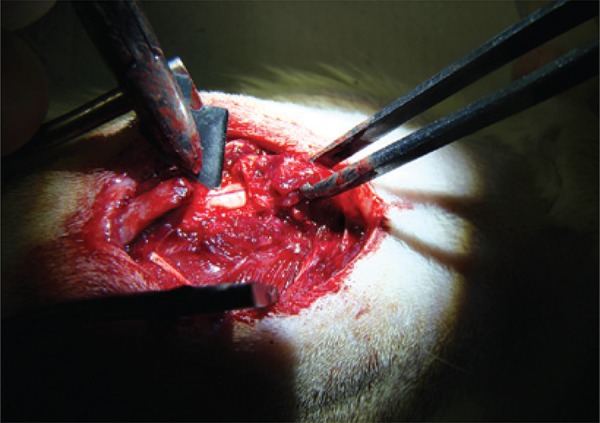
A 1mm incision in the dura mater with scalpel, causing lesion to the spinal cord involucrum

The same investigator operated on all the animals using a Carl Zeiss 3.5x surgical magnifier. The surgical site was exposed (Figure 1) by making a 2cm dorsal midline incision with a no. 15 scalpel blade to expose the vertebral spine from T8 to T12. The muscles inserted in the spinous processes were dissected and retracted using a bipolar coagulator, auto-static retractors, anatomical forceps, a perforating punch, a delicate Cobb rongeur, and a dura mater dissector. The dural sac was exposed by a laminectomy and a 5mm durotomy (Figure 2) was performed with a no. 15 scalpel blade, applied gently in the craniocaudal direction, and a 45^°^ angle up to the CSF leak, without damaging the subjacent neural tissue.

The repair of the dura mater was made with Prolene 6.0 sutures with an atraumatic needle, and with the aid of microdissection forceps, using simple separate stiches due to the small surgical incision.

**Figure 3:**
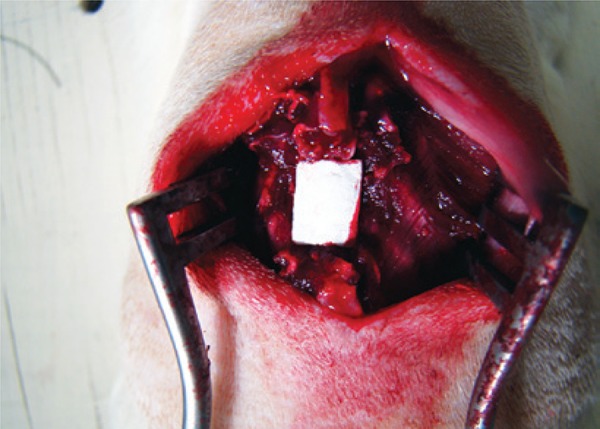
Placement of the silicon mesh over the spinal cord lesion

In DuraGen^®^ Group, Mesh Group, and Mesh Plus Suture Group, that were the groups with repair using mesh, 7mm squares of DuraGen^®^ (R) or silicone mesh (depending on the group), rough on one surface and smooth on the other, were used (Figure 3). The rough surface was in contact with the dura mater for the purpose of adhesion and isolation of the lesion.

An approximation of the tissue, muscle, fascia, and skin planes was performed using simple sutures of nylon monofilament 4,0. Postoperative analgesia was maintained for a period of 72 hours with meloxicam (0.2%, 2mg/kg), administered subcutaneously once a day.

The rats were returned to their original cages and the following parameters were monitored: (a) motor activity, evaluated from the gait pattern outside the cage, observing any paralysis and paresis, as well as their location and predominance; (b) healing of the surgical wound, observing for the presence of any inflammatory signs, such as erythema, heat, and humidity, and for the presence of either CSF fistulas or abscedation.

The animals were sacrificed 24 days after surgery by carbon dioxide inhalation, in accordance with the current legislation, and following the precepts of the *Conselho Nacional de Controle de Experimentação Animal* (CONCEA).

Following euthanasia, the spinal cord was removed *en bloc*, with a margin of 1cm at the edges, cephalic and caudal, in relation to the lesion site. The blocks were stored in bottles, identified by codes, with formaldehyde solution (10%), and sent to the Pathology Department of the *Faculdade de Medicina do ABC*.

### Histopathological analysis

Histopathological analysis was performed using slides, stained with hematoxylin-eosin (HE) and Masson trichrome, of the cranial, central, and caudal segments of the spinal cord, viewed under a binocular optical microscope. The evaluation was conducted by a single professional using the same Nikon microscope and observing and grading (absent, mild, moderate, and severe) the cases for inflammatory infiltrate, neovascularization and fibrosis.

Six levels of cuts were analyzed for each case, and the evaluation was concentrated on the posterior quadrants of the spinal cord. At each level, five consecutive fields were studied under 200-x magnification. The slides were stained with HE and the inflammatory infiltrate and density of the neovessels were evaluated semi-quantitatively and graded as absent (zero), mild (1), moderate (2), or severe (3). In the Masson trichrome, the degree of fibrous thickening of the dura mater was evaluated semi-quantitatively as absent (zero), mild (1), moderate (2), or severe (3). The pathologist was given no information about which group of rats the spinal cords belonged to (blind evaluation).

### Statistical analysis

A descriptive analysis of all study variables was conducted. The qualitative variables were presented as absolute and relative values, while the quantitative variables were presented in terms of their central tendency and dispersion values. For the variables that satisfied both of these principles, non-parametric tests (the Krusal-Wallis test) were used, at the level of alpha of 0.05, to compare the scores of the groups in relation to the variable, testing the hypotheses H0, if the scores obtained are independent from the group they belong to, and H1, if the scores obtained are dependent on the group they belong to.

## RESULTS

Table 1 describes the inflammatory infiltrate findings for all the rats (general) and by the groups of animals subjected to durotomy, sutures, DuraGen^®^, mesh, and mesh with sutures. Table 2 describes the same analysis, but for the neovascularization findings. Fibrosis is described in table 3 and table 4 describes the comparative statistical analysis among the groups for the three variables of inflammatory infiltrate, neovascularization, and fibrosis. All tables present the averages and standard deviations of the scores resulting from the histological analyses.

**Table 1:** Descriptive statistics of the inflammatory infiltrate in the five study groups

	Groups					General
	Durotomy	Suturas	DuraGen^®^	Mesh	Mesh + sutures	
Mean	1.3	0.5	2.0	1.0	0.5	1.1
Standard deviation	0.5	0.5	0.9	0.6	0.5	0.8
Coefficient of variation, %	38.8	109.6	44.7	63.2	109.6	77.4
Minimum	1	0	1	0	0	0
Maximum	2	1	3	2	1	3

**Table 2:** Descriptive statistics of neovascularization

	Group					General
	Durotomy	Sutures	DuraGen^®^	Mesh	Mesh + sutures	
Mean	1.3	1.0	1.8	1.3	0.7	1.2
Standard deviation	0.5	0.0	0.8	0.8	0.5	0.7
Coefficient of variation, %	38.8	0.0	41.1	61.4	77.0	55.2
Minimum	1	1.0	1	0	0	0
Maximum	2	1.0	3	2	1	3

**Table 3:** Descriptive statistics of fibrosis in the five groups evaluated

	Groups					General
	Durotomy	Sutures	DuraGen^®^	Mesh	Mesh + sutures	
Average	1.8	1.3	2.2	2.7	2.5	2.1
Standard deviation	0.8	0.5	0.8	0.5	0.5	0.8
Coefficient of variation, %	41.1	38.8	34.7	19.3	21.9	36.1
Minimum	1	1	1	2	2	1
Maximum	3	2	3	3	3	3

**Table 4:** Statistical analysis comparing the five study groups in relation to inflammatory infiltrate, neovascularization, and fibrosis

Groups	Inflammatory infiltrate		Neovascularization		Fibrosis	
	Result	p value	Result	p value	Result	p value
Durotomy *versus* simple suture	9.250	0.069	4.333	0.394	5.250	0.301
Durotomy *versus* DuraGen^®^	4.667	0.358	5.083	0.317	3.583	0.481
Durotomy *versus* Mesh	3.667	0.471	0.417	0.935	9.083	0.074
Durotomy *versus* mesh and suture	9.250	0.069	7.833	0.123	7.167	0.158
Simple sutures *versus* DuraGen^®^	13.917	0.006	9.417	0.064	8.833	0.082
Simple suture *versus* mesh	5.583	0.272	4.750	0.350	14.333	0.005
Simple suture *versus *mesh and suture	0.000	1.000	3.500	0.491	12.416	0.015
DuraGen^®^ *versus* mesh	8.333	0.101	4.667	0.358	5.500	0.279
DuraGen^®^ *versus* mesh and suture	13.917	0.006	12.917	0.011	3.583	0.481
Mesh *versus* mesh and suture	5.583	0.272	8.250	0.104	1.917	0.706

**Figure 4:**
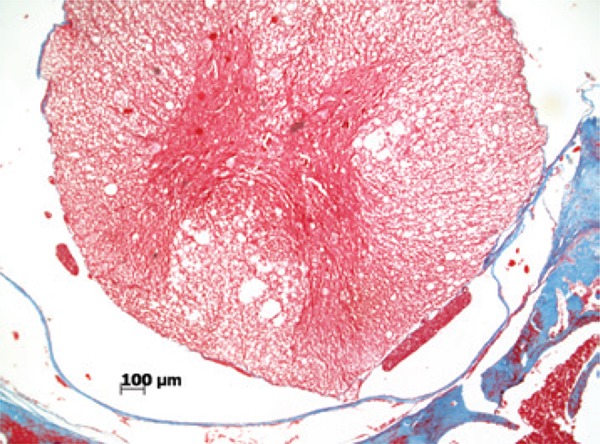
Photomicrography of a rat in Simple Suture Group (Masson trichrome, 40 X). Spinal cord is at the center, in red, enclosed by the pia mater and dura mater, stained in blue with a preserved aspect

**Figure 5:**
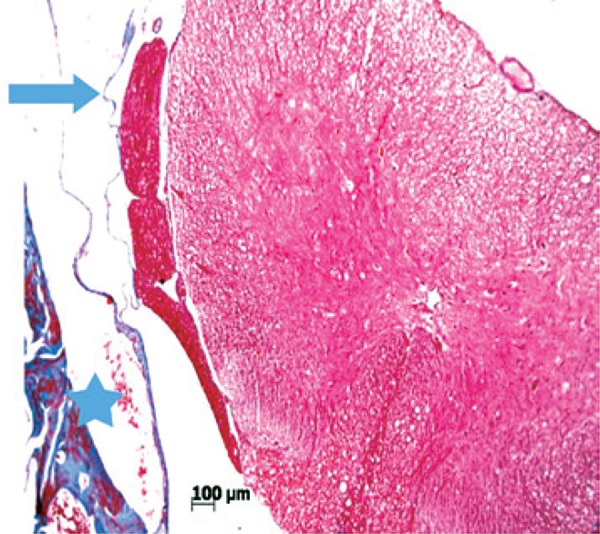
Photomicrography of a rat in DuraGen® Group (Masson trichrome, 100 X). Dura mater with normal thickness in the upper half (arrow) and thick in the lower half (star)

**Figure 6:**
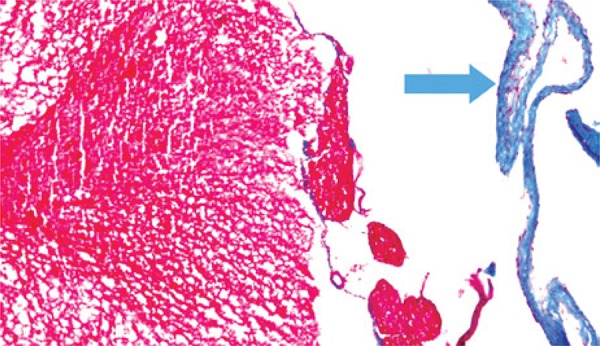
Photomicrography of a rat in Mesh Group (Masson trichrome, 200 X). The arrow indicates the fibroid thickening of the dura mater, stained in blue

In terms of histopathological aspects, when compared with the preserved aspects of the meninges (Figure 4), in relation to the presence of fibrosis, there was a statistically significant difference between the Simple Suture *versus* Mesh Groups (p=0.005) and between the Simple Suture *versus* Mesh Plus Suture Groups, showing that fibrosis is more evident when a foreign body (mesh or DuraGen^®^) is used to repair the lesion, as shown in figures 5 and 6.

**Figure 7:**
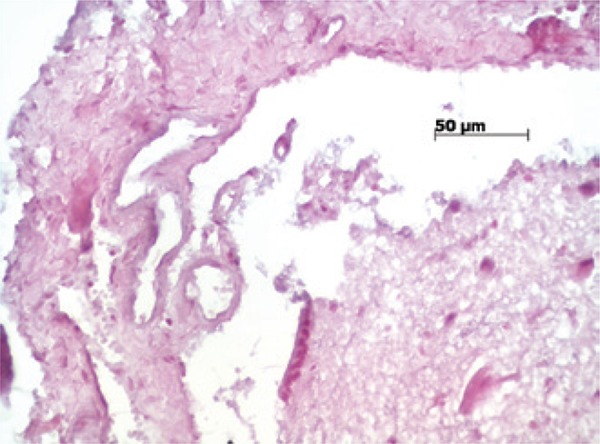
Photomicrography of a rat in Mesh Group (hematoxylin-eosin, 400x) indicated the presence of neovessels in the dura mater

In relation to the presence of neovessels (Figure 7), a statistically significant difference was noted between the DuraGen^®^ *versus* Mesh Plus Suture Groups (p=0.011). In relation to inflammatory infiltrate, there was a statistically significant difference between the Simple Suture *versus* DuraGen^®^ Groups (p=0.006).

## DISCUSSION

The spinal cord is enclosed in three fibrous membranes. The outermost is called the dura mater; the innermost, attached to the nervous system, is called the pia mater; and the middle one, the arachnoid. The phenomena that occur following a lesion to the dural membrane, at any level of the spinal cord, are still not completely understood. There is an insufficient number of publications, and most of those that do exist have small sample populations or a low number of cases.^(8)^ There is no consensus in the literature on the results achieved with the techniques, or the materials available on the market for dural repair. Additional research needs to be conducted to develop better experimental models of dural lesion in animals, to establish and standardize evaluation methods, and to continuously compare new dural repair techniques and materials.

The search for better forms of treatment for dural lesions requires the development of experimental models, in which the new techniques developed for human use can be reproduced, and both the effects caused by the implants and the reactions produced in the dural and spinal cord tissues can be evaluated.

There are numerous animal experimentation models for the development of dural repair techniques and materials, for testing the safety, efficacy, efficiency, and effectiveness of the implant materials, and for evaluating spinal cord degeneration in experimental models. Many investigators employ experimental models using rats. Rabbits are also used^(13)^ and there are experimental models that use larger animals, such as dogs,^(8)^ pigs,^(12)^ and primates.^(14)^ However, we decided to use Wistar rats of the Wistar breed due to the availability and quality of the animals, and the ease of handling and housing following surgery.

The surgical technique used in our study was a dural lesion in the thoracic region, even though most authors use the lumbar region^(8,15)^ and the cranial region^(14)^ as the sites of choice for conducting tests with dural substitutes. The option to produce a dural lesion by performing a laminectomy in the thoracic region was chosen because it is easier to identify possible iatrogenic lesions of the spinal cord caused by the surgical technique through clinical parameters, enabling the exclusion of animals with any motor changes. We also chose the thoracic level since the vast majority of iatrogenic lesions occur at lumbar level, and lesions with dural involvement, in the case of fractures, occur with equal frequency in the lumbar and thoracic regions.^(15,16)^

In our study, in order to evaluate the efficacy of dural repair using silicone mesh, we compared the dural repair techniques and commercial materials that are most often mentioned and tested in the medical literature,^(17)^ safe (contamination and reaction of the host tissue), and available in Brazil. We aimed to compare dural repair techniques using simple sutures, collagen membrane, and silicone mesh.

Dural suture is a well-established method. It can be combined with other methods. However, it has extremely variable closure techniques. The type, diameter, and material of the suture thread, and the suture technique used (number, spacing, and type of stitch) can vary. Isolated, continuous simple, or continuous anchored sutures are used.^(3)^ The study by Megyesi et al. demonstrated greater efficacy of the simple-type suture with isolated stitches in *in vitro* tests to evaluate the resistance of the dura mater closure when submitted to hydrostatic pressure, and this closure option was used in our study.^(18)^ There is also a great possibility of variation in the suture thread used. The sutures most often used are polyamide monofilament (nylon) 4-0, Prolene 6-0, and silk 3-0. Prolene 6-0 thread with an atraumatic needle was used for the dural sutures. Closure was achieved with three to four isolated simple stitches, with spacing of approximately 2mm between them, due to the small size of the neural structures.

DuraGen^®^ bovine collagen membrane was chosen because of its viability and easy of use. It is not sutured to the dura mater (the material is not suturable); its fixation is achieved by placing it directly on the lesion site and applying drops of 9% NaCl saline solution. The use of DuraGen^®^ as a substitute or sealant for dural defects is considered to be safe in terms of contamination and reactions of the host tissue.^(17)^

In our study, we compared two groups (sutures and DuraGen^®^) with the silicone mesh with and without fixation with sutures, and observed that as to the inflammatory infiltration, the silicone mesh caused less of an inflammatory reaction, on average, than DuraGen^®^, and a similar reaction to that of sutures. Knowing that in some cases, simple sutures are not feasible for the closure of dura mater due to the large size of the lesion, both silicone mesh and DuraGen^®^ can be options for replacement of the membrane surrounding the spinal cord. Concerning neovascularization, there was a statistically significant difference between the DuraGen^®^ and Silicone Mesh Plus Suture Groups. The results of Silicone Mesh Plus Suture were significantly different from those of DuraGen^®^ in terms of inflammatory infiltrate and neovascularization, and different from simple sutures as to fibrosis.

The main limitations of our study were as follows: the possible different timing of the scar tissue process comparing the animal model to humans and, secondly, the short post-operative period of observation before the rats became sacrificed. We found no studies detailing the late results of experimental silicone mesh repair and neither scientific data about the costs involved with this technique. More studies including meta-analysis and systematic reviews are necessary to better evaluate, compare and confirm these results proving the value of silicone mesh in dural lesions difficult to repair.

## CONCLUSIONS

When compared to other similar commercially-used products, silicone mesh showed potential for use as a dura mater protector, but further studies are necessary to confirm these initial results.
