# Supplementary material for: Histological analysis of the repair of dural lesions with silicone mesh in rats subjected to experimental lesions
Source: Einstein (Sao Paulo). 2015 Oct-Dec;13(4):567–73. doi: 10.1590/S1679-45082015AO3378 (PMC4878632; doi:10.1590/S1679-45082015AO3378)
Supplement: Translation

# Translation: Análise histológica do reparo de lesões da dura com tela de silicone em ratos submetidos à lesão experimental

**Authors:** Fernando William Figueiredo da Rosa, Pedro Henrique Isoldi Pohl, Ana Maria Amaral Antônio Mader, Carla Peluso de Paiva, Aline Amaro dos Santos, Bianca Bianco, Luciano Miller Reis Rodrigues

**Affiliations:** 1 Faculdade de Medicina do ABC, Santo André, SP, Brasil.

**Keywords:** Dura-Máter, Meninges, Procedimentos cirúrgicos operatórios, Cirurgia torácica, Silício, Modelos animais

## Abstract

**Objetivo:**

: Avaliar reação inflamatória, fibrose e neovascularização da reparação da lesão dural em ratos Wistar, comparando quatro diferentes técnicas: pontos simples, membrana de colágeno bovino, tela de silicone e tela de silicone associada a pontos simples.

**Métodos:**

: Trinta ratos Wistar foram randomizados em cinco grupos: o primeiro foi um grupo controle, submetido somente à durotomia. Os outros também foram submetidos à durotomia, porém sofreram sutura simples, reparo com membrana de colágeno bovino, tela de silicone e tela de silicone com sutura. Os animais foram sacrificados, e a coluna foi submetida à avaliação histológica com um escore (variando de zero a 3) para inflamação, neovascularização e fibrose.

**Resultados:**

: A fibrose foi significativamente diferente, comparando-se sutura simples e tela de silicone (p=0,005) e sutura simples e tela com fio de sutura (p=0,015), demonstrando que a fibrose foi mais intensa quando um corpo estranho foi utilizado na reparação. Membrana bovina foi significativamente diferente da tela mais sutura (p=0,011) em relação à vascularização. A inflamação foi significativamente diferente entre os grupos submetidos à sutura simples e ao reparo com membrana de colágeno bovino.

**Conclusão:**

: A tela de silicone, comparada com produtos similares com disponibilidade comercial, é uma possível alternativa como protetor de dura-máter. Mais estudos são necessários para comprovar esses resultados.

## INTRODUÇÃO

As causas mais comuns de lesões durais são trauma espinal e lesões iatrogênicas.^(1-5)^ As fraturas vertebrais podem causar lesão dural^(3-5)^ em até 19% dos casos, quando os elementos espinais posteriores também sofrem fraturas associadas a fraturas em explosão do corpo vertebral.^(4,5)^ Nos procedimentos lombares, há prevalência de 1 a 17% de lesões acidentais da dura-máter.^(1,2)^ Várias estruturas autólogas e materiais sintéticos têm sido utilizados na reparação da dura-máter, incluindo gordura autóloga, fáscia lata, dura-máter liofilizada e materiais sintéticos, com grande variabilidade nos resultados clínicos.^(6-8)^

O aumento no número de indicações e na complexidade dos procedimentos cirúrgicos espinais resultaram numa maior prevalência de lesões durais,^(3,9)^ particularmente nas cirurgias reoperatórias.^(1,3)^ A falta de atenção aos princípios da cirurgia moderna, a complexidade dos casos, a falta de experiência e a pressa para finalizar o procedimento cirúrgico são fatores que podem contribuir para essas estatísticas. Nos Estados Unidos, o aumento na frequência das laminectomias lombares para doenças degenerativas dos discos intervertebrais foi a principal causa de lesões durais, que ocorrem geralmente no momento da abertura do ligamento amarelo.^(10)^

A maioria das lesões é reconhecida no intraoperatório. Em alguns casos, nos quais a lesão é incompleta, afetando apenas a camada externa da dura-máter sem atingir a aracnoide, pode não haver extravasamento liquórico imediato, mas isso pode vir a ocorrer no pós-operatório.^(3)^ De acordo com Wang et al., o reparo intraoperatório imediato não aumenta a morbidade perioperatória nem altera os resultados finais.^(1)^

Nas cirurgias de reparação da dura-máter, um aspecto importante é a prevenção de aderências pós-operatórias.^(9)^ Geralmente, o substituto dural utilizado é a dura-máter liofilizada de cadáveres, como também ocorre nos casos de reparo de defeitos durais e meningomieloceles, mas há a desvantagem do risco de transmissão de doenças infecciosas.^(9)^

O reparo dural aumenta o tempo total de cirurgia em aproximadamente 20 a 30 minutos.^(1)^ A técnica adequada deve ser de rápida e de fácil aplicação,^(3)^ mas as lesões localizadas na região anterior ou lateral podem ser mais complicadas, exigindo outros tipos de técnicas.^(3,4)^ Alguns autores recomendam uma combinação de métodos.^(1-3)^

As lesões durais têm sido tratadas com sucesso por meio de reparo primário, seguido de repouso no leito em decúbito dorsal.^(1,3,4)^ Várias formas de reparo foram usadas, como suturas com pontos separados,^(1,5)^ suturas contínuas,^(3)^ enxertos autólogos de fáscia,^(3)^ selante de fibrina^(11)^ e matriz de colágeno porcino.^(12)^ O silicone tem sido usado em diversas áreas cirúrgicas, com isso desenvolvemos uma membrana de silicone para avaliarmos a aplicabilidade nas lesões durais.

É muito difícil comparar os diferentes tipos de tratamento das lesões durais, porque os poucos estudos existentes foram realizados com base em um número pequeno de pacientes.^(1)^ Este estudo é importante devido à falta de consenso na literatura internacional sobre os métodos de reparação e os novos materiais que surgem para seu tratamento. Além disso, os estudos experimentais controlados são raros e ajudam a comparar aspectos fundamentais, como inflamação, biocompatibilidade, neovascularização e fibrose nos diferentes métodos de reparação dural.

## OBJETIVO

Avaliar o potencial do uso da tela de silicone no reparo de lesões da dura-máter e comparar com várias outras técnicas.

## MÉTODOS

Este estudo experimental com ratos foi realizado no Laboratório de Experimentação Animal e Biotério da Faculdade de Medicina do ABC (FMABC), de acordo com a legislação relevante, tendo sido aprovado pelo Comitê de Ética em Experimentação Animal da FMABC (protocolo número 003/2010).

Foram utilizados 30 ratos machos Wistar (*Rattus norvergicus*), com pelo menos 12 semanas de idade, pesando de 300 a 350g, alojados em grupos de até três animais por gaiola. Os ratos foram mantidos em condições ambientais (manuseio, *ad libitum*, troca periódica da forração de serragem e limpeza da gaiola) a uma temperatura controlada de aproximadamente 28^°^C, com fotoperíodo de 12 horas de claro e 12 horas de escuro, e alimentação regular diária com ração adequada para a espécie, até o final da experiência.

Os ratos foram avaliados no tocante a condições gerais, peso e habilidades motoras. Estabeleceram-se os seguintes critérios de exclusão: óbito após a lesão; perda de tecido na área da lesão; anomalias observadas macroscopicamente na região medular; ausência de controle esfincteriano anal ou vesical; ausência de atividade motora após a lesão; e perda de peso superior a 20% do peso do animal.

Os ratos foram separados aleatoriamente (por sorteio) em cinco grupos de seis animais cada e identificados na cauda. Todos foram submetidos a lesão da dura-máter. Os grupos foram os seguintes Grupo Durotomia, controle, com ratos submetidos à lesão dural sem tratamento subsequente; Grupo Sutura Simples, com lesão dural reparada com sutura simples; Grupo DuraGen^®^, com lesão dural reparada com substituto dural de colágeno bovino (DuraGen^®^) como proteção, sem fixação; Grupo Tela, com lesão dural reparada com tela de silicone como proteção, sem fixação; Grupo Tela e Sutura com lesão da dura-máter reparada com sutura simples e tela de silicone como proteção.

### Descrição da técnica cirúrgica

Utilizou-se anestesia intraperitoneal (solução de quetamina, 50 a 80mg/kg, com cloridrato de xilazina a 2%, 10mg/kg, e atropina, 0,05mg/kg). Confirmou-se a anestesia profunda pela ausência de reflexo corneal e ausência de reação à dor profunda infligida por compressão interdigital das patas. Utilizou-se cefalotina subcutânea (25mg/kg) para terapia antibiótica profilática após a anestesia, 24 e 48 horas após a produção da lesão. Nos ratos que apresentaram infecção, o tratamento foi prolongado até o décimo dia, e nos casos de infecção persistente, o animal foi sacrificado (critério de exclusão).

O mesmo investigador operou todos os animais usando uma lupa cirúrgica Carl Zeiss com 3,5x de aumento. O sítio cirúrgico foi exposto (Figura 1) por meio de uma incisão dorsal de 2cm na linha média com uma lâmina de bisturi 15, para expor a coluna vertebral de T8 a T12. Os músculos inseridos nos processos espinhosos foram dissecados e afastados, utilizando-se um coagulador bipolar, afastadores autoestáticos, pinças anatômicas, um *punch*, uma rugina Cobb delicada e um descolador de dura-máter. O saco dural foi exposto por laminectomia, sendo, então, realizada uma durotomia de 5mm (Figura 2) com lâmina de bisturi 15, aplicada gentilmente na direção craniocaudal, em ângulo de 45° até o extravasamento liquórico, sem danificar o tecido neural subjacente.

**Figura 1:** Exposição cirúrgica da medula espinal

**Figura 2:** Incisão de 1mm na dura-máter com bisturi, causando lesão no invólucro da medula espinal

O reparo da dura-máter foi feito com suturas de Prolene 6.0 com agulha atraumática, com auxílio de pinça de microdissecção, utilizando-se pontos separados simples, devido ao reduzido tamanho da incisão cirúrgica.

Nos grupos DuraGen^®^, Tela e Tela e Sutura, que foram os grupos com reparo usando tela, foram utilizados quadrados de 7mm de DuraGen^®^ (R) ou tela de silicone (dependendo do grupo), com uma superfície áspera e a outra lisa (Figura 3). A superfície áspera ficou em contato com a dura-máter, para fins de aderência e isolamento da lesão.

**Figura 3:** Colocação da tela de silicone sobre a lesão da medula espinal

Realizou-se a aproximação dos planos de tecido, músculo, fáscia e pele, utilizando-se suturas simples com fio de nylon monofilamento 4.0. Manteve-se analgesia pós-operatória por um período de 72 horas com meloxicam (0,2%, 2mg/kg), administrado por via subcutânea, uma vez por dia.

Os ratos foram levados de volta para suas gaiolas originais, e os seguintes parâmetros foram monitorados: (a) atividade motora, avaliada pelo padrão de marcha fora da gaiola, observando-se quaisquer paralisias ou paresias, bem como sua localização e predominância; (b) cura da ferida cirúrgica, observando-se a presença de quaisquer sinais flogísticos, tais como eritema, calor e umidade, bem como a presença de outras fístulas liquóricas ou abscedação.

Os animais foram sacrificados 24 dias após a cirurgia por inalação de dióxido de carbono, de acordo com a legislação vigente, e seguindo os preceitos do Conselho Nacional de Controle de Experimentação Animal (CONCEA).

Após a eutanásia, a medula espinal foi removida em bloco, com uma margem de 1cm nas bordas, cefálica e caudal, em relação ao sítio da lesão. Os blocos foram guardados em frascos, identificados por códigos, com solução de formaldeído (10%), e enviados para o Serviço de Anatomia Patológica da Faculdade de Medicina do ABC.

### Análise histopatológica

A análise histopatológica foi realizada utilizando-se lâminas coradas com hematoxilina-eosina (HE) e tricromo de Masson dos segmentos craniano, central e caudal da medula espinal, visualizadas em microscópio óptico binocular. A avaliação foi realizada por um único profissional, que utilizou o mesmo microscópio Nikon e observou e classificou os casos (ausente, leve, moderado e grave) em relação a infiltrado inflamatório, neovascularização e fibrose.

Seis níveis de corte foram analisados para cada caso, e a avaliação concentrou-se nos quadrantes posteriores da medula espinal. Em cada nível, cinco campos consecutivos foram estudados com aumento de 200 vezes. As lâminas foram coradas com HE, e o infiltrado inflamatório e a densidade dos neovasos foram avaliados semiquantitativamente e classificados como ausente (zero), leve (1), moderado (2) ou severo (3). No tricromo de Masson, o grau de espessamento fibroso da dura-máter foi avaliado em termos semiquantitativos, como ausente (zero), leve (1), moderado (2) ou grave (3). O patologista não recebeu nenhuma informação referente ao grupo de ratos ao qual pertenciam as medulas espinais (avaliação cega).

### Análise estatística

Realizou-se análise descritiva de todas as variáveis do estudo. As variáveis qualitativas foram apresentadas como valores absoluto e relativo, ao passo que as variáveis quantitativas foram apresentadas em termos de sua tendência central e valores de dispersão. Para as variáveis que satisfaziam ambos princípios, utilizaram-se testes não paramétricos (o teste Krusal-Wallis), considerando-se alfa de 0,05, para comparar a classificação dos grupos em relação à variável, testando as hipóteses H0, se as classificações obtidas forem independentes do grupo a que pertencem, e H1, se as classificações obtidas forem dependentes do grupo a que pertencem.

## RESULTADOS

A tabela 1 descreve os achados de infiltrado inflamatório para todos os ratos (geral) e por grupos de animais submetidos a durotomia, suturas, DuraGen^®^, tela e tela com suturas. A tabela 2 descreve a mesma análise, mas para os achados de neovascularização. A fibrose é descrita na tabela 3, e a tabela 4 descreve a análise estatística comparativa entre os grupos para as três variáveis (infiltrado inflamatório, neovascularização e fibrose). Todas as tabelas apresentam as médias e os desvios padrão das classificações obtidas a partir da análise histológica.

**Tabela 1:** Estatística descritiva do infiltrado inflamatório em cinco grupos de estudo

	Grupos					Geral
	Durotomia	Suturas	DuraGen^®^	Tela	Tela + suturas	
Média	1,3	0,5	2,0	1,0	0,5	1,1
Desvio padrão	0,5	0,5	0,9	0,6	0,5	0,8
Coeficiente de variação, %	38,8	109,6	44,7	63,2	109,6	77,4
Mínimo	1	0	1	0	0	0
Máximo	2	1	3	2	1	3

**Tabela 2:** Estatística descritiva de neovascularização

	Grupo					Geral
	Durotomia	Suturas	DuraGen^®^	Tela	Tela + suturas	
Média	1,3	1,0	1,8	1,3	0,7	1,2
Desvio padrão	0,5	0,0	0,8	0,8	0,5	0,7
Coeficiente de variação, %	38,8	0,0	41,1	61,4	77,0	55,2
Mínimo	1	1,0	1	0	0	0
Máximo	2	1,0	3	2	1	3

**Tabela 3:** Estatística descritiva de fibrose nos cinco grupos avaliados

	Grupos					Geral
	Durotomia	Suturas	DuraGen^®^	Tela	Tela + suturas	
Média	1,8	1,3	2,2	2,7	2,5	2,1
Desvio padrão	0,8	0,5	0,8	0,5	0,5	0,8
Coeficiente de variação, %	41,1	38,8	34,7	19,3	21,9	36,1
Mínimo	1	1	1	2	2	1
Máximo	3	2	3	3	3	3

**Tabela 4:** Análise estatística comparando os cinco grupos de estudo em relação a infiltrado inflamatório, neovascularização, e fibrose

Grupos	Infiltrado inflamatório		Neovascularização		Fibrose	
	Resultado	Valor de p	Resultado	Valor de p	Resultado	Valor de p
Durotomia *versus* suturas simples	9,250	0,069	4,333	0,394	5,250	0,301
Durotomia *versus* DuraGen^®^	4,667	0,358	5,083	0,317	3,583	0,481
Durotomia *versus* tela	3,667	0,471	0,417	0,935	9,083	0,074
Durotomia *versus *tela e suturas	9,250	0,069	7,833	0,123	7,167	0,158
Suturas simples *versus* DuraGen^®^	13,917	0,006	9,417	0,064	8,833	0,082
Suturas simples *versus* tela	5,583	0,272	4,750	0,350	14,333	0,005
Suturas simples *versus* tela e suturas	0,000	1,000	3,500	0,491	12,416	0,015
DuraGen^®^ *versus* tela	8,333	0,101	4,667	0,358	5,500	0,279
DuraGen^® ^ *versus* tela e suturas	13,917	0,006	12,917	0,011	3,583	0,481
Tela *versus* tela e suturas	5,583	0,272	8,250	0,104	1,917	0,706

Em termos dos aspectos histopatológicos, quando comparados com os aspectos preservados das meninges (Figura 4), no tocante à presença de fibrose, houve diferença estatisticamente significativa entre o Grupo Suturas Simples *versus* o Grupo Tela (p=0,005) e entre o Grupo Suturas simples *versus* o Grupo Tela com Suturas, mostrando que a fibrose foi mais evidente quando um corpo estranho (tela ou DuraGen^®^) foi usado para reparar a lesão, como mostrado nas figuras 5 e 6.

**Figura 4:** Fotomicrografia de um rato do Grupo Sutura Simples (tricromo de Masson, 40X). A medula espinal está no centro, em vermelho, envolta pela pia-máter e a dura-máter, corada em azul com aspecto preservado

**Figura 5:** Fotomicrografia de um rato do Grupo DuraGen® (tricromo de Masson, 100X). Dura-máter com espessura normal na metade superior (seta) e espessada na metade inferior (estrela)

**Figura 6:** Fotomicrografia de um rato do Grupo Tela (tricromo de Masson, 200X). A seta indica espessamento fibroide da dura-máter corado em azul

Em relação à presença de neovascularização (Figura 7), observou-se uma diferença estatisticamente significativa entre o grupo DuraGen^®^ e o Grupo Tela e Sutura (p=0,011). Em relação ao infiltrado inflamatório, houve diferença estatisticamente significativa entre o Grupo Sutura Simples e o Grupo DuraGen^®^ (p=0,006).

**Figura 7:** A fotomicrografia de um rato do Grupo Tela (hematoxilina-eosina, 400x) indicou a presença de neovascularização na dura-máter

## DISCUSSÃO

Três membranas fibrosas envolvem a medula espinal. A mais externa chama-se dura-máter; a mais interna, anexa ao sistema nervoso, chama-se pia-máter; e a do meio, aracnoide. Os fenômenos que ocorrem após a lesão da membrana dural, em qualquer nível da medula espinal, ainda não estão completamente esclarecidos. Há um número insuficiente de publicações, e a maioria das que existem referem-se a uma pequena amostra da população ou têm pequeno número de casos.^(8)^ Não há consenso na literatura sobre os resultados obtidos com as técnicas ou os materiais disponíveis no mercado para reparação dural. É preciso que sejam realizadas mais pesquisas para desenvolver melhores modelos experimentais de lesão dural em animais, estabelecer e padronizar os métodos de avaliação, e comparar continuamente novas técnicas e materiais de reparação dural.

A pesquisa de melhores formas de tratamento de lesões durais exige o desenvolvimento de modelos experimentais, em que novas técnicas desenvolvidas para uso humano possam ser reproduzidas, e tanto os efeitos causados pelos implantes quanto as reações teciduais produzidas na dura-máter e na medula espinal possam ser avaliados.

Há vários modelos de experimentação animal para desenvolver técnicas e materiais de reparação dural, para testar a segurança, a eficácia, a eficiência e a resolutividade dos materiais de implante, além de avaliar a degeneração da medula espinal nos modelos experimentais. Muitos pesquisadores utilizaram modelos experimentais com ratos. Também foram utilizados coelhos^(13)^ e há modelos experimentais que utilizam animais maiores, como cães,^(8)^ porcos^(12)^ e primatas.^(14)^ No entanto, decidimos usar ratos da raça Wistar devido à disponibilidade e à qualidade dos animais, e por causa da facilidade de manuseio e alojamento após a cirurgia.

A técnica cirúrgica utilizada em nosso estudo foi uma lesão dural na região torácica, embora a maioria dos autores utilize as regiões lombar^(8,15)^ e cranial^(14)^ como locais de escolha para a realização de testes com substitutos durais. A opção de produzir uma lesão dural por meio de laminectomia na região torácica foi escolhida por ser mais fácil identificar possíveis lesões iatrogênicas da medula espinal causadas pela técnica cirúrgica por meio de parâmetros clínicos, permitindo a exclusão de animais com alterações motoras. Também escolhemos o nível torácico porque a maioria das lesões iatrogênicas ocorre no nível lombar, e as lesões com envolvimento dural, no caso de fraturas, ocorrem com igual frequência na região lombar e na região torácica.^(15,16)^

Em nosso estudo, para avaliação da eficácia do reparo dural usando tela de silicone, comparamos as técnicas de reparação dural e os materiais comerciais mais frequentemente citados e testados na literatura médica,^(17)^ seguros (em relação a contaminação e reação do tecido hospedeiro) e disponíveis no Brasil. Procuramos comparar as técnicas de reparação dural que usam suturas simples, membrana de colágeno e tela de silicone.

A sutura dural é um método bem estabelecido, que pode ser combinado com outros métodos. No entanto, tem técnicas extremamente variáveis de fechamento. Tipo, diâmetro e material do fio de sutura, e a técnica de sutura utilizada (número, espaçamento e tipo de ponto) podem variar. Foram utilizadas suturas isoladas, sutura contínua simples ou sutura contínua ancorada.^(3)^ O estudo de Megyesi et al. demonstrou maior eficácia da sutura tipo simples com pontos isolados nos testes *in vitro *para avaliação da resistência do fechamento da dura-máter, quando submetida à pressão hidrostática, e essa foi a opção de fechamento utilizada em nosso estudo.^(18)^ Há também grande possibilidade de variação no fio de sutura utilizado. Com maior frequência, as suturas mais utilizadas são monofilamento de poliamida (nylon) 4-0, Prolene 6-0 e seda 3-0. O fio de Prolene 6-0 com agulha atraumática foi utilizado para as suturas durais. O fechamento foi efetuado com três a quatro pontos isolados simples, com espaçamento de aproximadamente 2mm entre eles, devido ao reduzido tamanho das estruturas neurais.

Foi escolhida a membrana de colágeno bovino DuraGen^®^ por sua viabilidade e facilidade de uso. Ela não é suturada à dura-máter (o material não é suturável). Sua fixação é feita colocando-se a membrana diretamente sobre o sítio da lesão e aplicando-se gotas de solução fisiológica de NaCl a 9%. O uso de DuraGen^®^ como substituto ou selante para defeitos durais é considerado seguro em termos de contaminação e de reações do tecido hospedeiro.^(17)^

Em nosso estudo, comparamos dois grupos (suturas e DuraGen^®^) com a tela de silicone com e sem fixação por meio de suturas, e observamos que, em relação ao infiltrado inflamatório, a tela de silicone causou, em média, menos reação inflamatória do que o DuraGen^®^ e apresentou reação semelhante à das suturas. Sabendo que em alguns casos as suturas simples não são praticáveis para o fechamento da dura-máter devido ao grande tamanho da lesão, tanto a tela de silicone quanto o DuraGen^®^ podem ser opções para a substituição da membrana que envolve a medula espinal. Quanto à neovascularização, houve uma diferença estatisticamente significativa entre o Grupo DuraGen^®^ e o Grupo Tela de Silicone com suturas. Os resultados do Grupo Tela de Silicone com suturas foram significativamente diferentes daqueles do Grupo DuraGen^®^, em termos de infiltrado inflamatório e neovascularização, e diferentes do Grupo Sutura Simples, em relação à fibrose.

As principais limitações de nosso estudo foram a possível diferença do curso de tempo no processo de cicatrização nos modelos animal e humano e, em segundo lugar, o curto período de observação pós-operatório antes de os ratos serem sacrificados. Não encontramos nenhum estudo que detalhasse os resultados tardios do reparo experimental com tela de silicone, ou mostrasse dados científicos sobre os custos envolvidos nessa técnica. Mais estudos que incluam metanálises e revisões sistemáticas são necessários para avaliar melhor, comparar e confirmar esses resultados, comprovando o valor da tela de silicone nas lesões durais de difícil reparo.

## CONCLUSÃO

Quando comparada com outros produtos comerciais de uso semelhante, a tela de silicone mostrou potencial em seu uso como protetor da dura-máter, porém mais estudos são necessários para confirmar esses resultados iniciais.
